# Postexercise immune-inflammatory improvement in type 2 diabetes is associated with baseline insulin resistance

**DOI:** 10.3389/fendo.2025.1679142

**Published:** 2025-11-18

**Authors:** Wei Cheng, Kai Guo, Xuelian Zhang, Keqin Zhang

**Affiliations:** 1 Department of Endocrinology, Yangpu Hospital, School of Medicine, Tongji University, Shanghai, China; 2 Department of Endocrinology and Metabolism, Tongji Hospital, School of Medicine, Tongji University, Shanghai, China

**Keywords:** systemic immune-inflammation index, type 2 diabetes mellitus, insulin resistance, exercise intervention, heterogeneous response

## Abstract

**Purpose:**

To examine how baseline insulin resistance (IR) modulates exercise-induced changes in systemic immune-inflammation index (SII) and metabolic parameters in type 2 diabetes mellitus (T2DM).

**Methods:**

Fifty-five T2DM patients stratified by fasting C-peptide tertiles into low- (Group 1), moderate- (Group 2), and high-IR groups (Group 3) completed a 4-week moderate-intensity combined aerobic-resistance exercise program. Changes in SII (neutrophils × platelets/lymphocytes), anthropometrics, and glucolipid markers were assessed, with ANCOVA and hierarchical regression modeling intergroup differences and predictors (Clinical Trial Registration ID: ChiCTR2200066710).

**Results:**

Significant reductions in weight, BMI, and body fat% occurred in Group 1/Group 2 (all *p*<0.05) but not Group 3. SII decreased in Group 1 (*p*<0.05) yet increased in Group 3 due to neutrophil elevation (*p*<0.05). Fasting glucose and HbA1c improved across all groups (*p*<0.05), with Group 3’s glycemic benefits independent of weight loss or anti-inflammatory effects. Baseline C-peptide independently predicted increases in ΔSII across all adjusted models (β=19.85–21.94, *p*<0.01), whereas covariates including age, diabetes duration, and BMI showed no significant effects.

**Conclusion:**

Severe baseline IR attenuates exercise-mediated SII improvement and body composition optimization, whereas glycemic benefits remain IR-independent, necessitating IR-stratified exercise prescriptions.

**Clinical trial registration:**

Chinese Clinical Trial Registry, identifier ChiCTR2200066710.

## 1 Introduction

Chronic inflammation is recognized as a core pathological basis for the development of multiple chronic diseases, arising from complex interactions among social, environmental, and behavioral factors (1). Systemic chronic inflammation (SCI), through sustained activation of immune response pathways, constitutes a shared risk factor for major noncommunicable diseases including cardiovascular disease, malignancy, type 2 diabetes mellitus (T2DM) (2). Substantial evidence confirms that immune cells—such as macrophages, neutrophils, monocytes, and platelets—participate in systemic inflammatory responses and contribute to T2DM progression and its complications (3). Upon activation, these cells produce proinflammatory cytokines (e.g., IL-8, IL-6, IL-1β), which have been documented to correlate with adverse clinical outcomes in diabetes, including cardiovascular events and nephropathy (4–6).

The molecular mechanisms through which systemic inflammation promotes the development and progression of T2DM are increasingly being elucidated. At the cellular level, key inflammatory signaling pathways, particularly the nuclear factor kappa-B (NF-κB) and c-Jun N-terminal kinase (JNK) pathways, have been identified as central connectors linking inflammation to insulin resistance (7–12). In metabolic tissues such as adipose tissue, liver, and skeletal muscle, nutrient excess and metabolic stress trigger the activation of immune cells (e.g., a shift to M1 macrophages) and the production of pro-inflammatory cytokines like tumor necrosis factor-alpha (TNF-α) and interleukin-1beta (IL-1β) (13–16). These cytokines, in turn, can directly impair insulin signaling by inducing serine phosphorylation of insulin receptor substrate (IRS) proteins, which suppresses their normal tyrosine phosphorylation and impedes the downstream translocation of glucose transporter 4 (GLUT4), ultimately leading to cellular glucose uptake deficiency (17, 18). Concurrently, within pancreatic islets, these systemic inflammatory mediators can induce β-cell apoptosis and dysfunction, further compromising insulin secretion and establishing a vicious cycle that propels the progression from insulin resistance to overt diabetes (19, 20). Therefore, targeting these underlying inflammatory processes presents a promising therapeutic strategy for T2DM management.

With advances in inflammation biology, peripheral blood inflammatory biomarkers have gained increasing clinical relevance. Among these, the Systemic Immune-Inflammation Index (SII)—a novel composite metric integrating neutrophil, lymphocyte, and platelet counts—provides a comprehensive assessment of immune-inflammatory homeostasis. Its unique capability to evaluate the global regulatory effects of systemic interventions (e.g., exercise) on inflammatory networks has garnered significant attention in disease risk prediction and monitoring (21–24).

Insulin resistance (IR) represents a fundamental pathological mechanism in T2DM (25, 26). Although the precise molecular pathways underlying IR remain incompletely defined, compelling evidence establishes a bidirectional interplay between systemic inflammation and IR, wherein immune dysregulation plays a pivotal pathogenic role (27, 28). While the hyperinsulinemic-euglycemic clamp remains the gold standard for IR quantification, its technical complexity and high cost limit clinical applicability (29). Consequently, fasting C-peptide - an equimolar byproduct of proinsulin cleavage - serves as our preferred biomarker due to its extended half-life (20–30 min vs. insulin’s 3–5 min), absence of hepatic first-pass metabolism, and superior reliability in evaluating endogenous insulin secretion (30, 31). Leveraging the heterogeneity of IR in T2DM, we innovatively implemented a C-peptide-based stratification strategy to investigate differential responses to exercise interventions.

Exercise therapy is recommended as first-line management for T2DM in international guidelines. However, existing research predominantly focuses on glucolipid metabolic improvements, leaving significant gaps in understanding its systemic anti-inflammatory mechanisms. Notably, although both aerobic and resistance training demonstrate independent anti-inflammatory benefits (32–34), the combined effects of these modalities on SII remain systematically unexamined in IR-stratified T2DM populations. This study therefore implements a 4-week combined aerobic-resistance training intervention. Through longitudinal tracking of SII dynamics, we aim to quantify regulatory effects of combined exercise on systemic immune-inflammation (measured by SII), determine their modulation by baseline metabolic determinants (C-peptide levels, IR severity, HbA1c/FPG), and generate evidence for optimizing T2DM exercise prescriptions.

## 2 Materials and methods

### 2.1 Study participants

A total of 68 adults with T2DM were consecutively recruited from the Department of Endocrinology, Yangpu District Central Hospital (Shanghai, China) between March and October 2024. All participants provided written informed consent after comprehensive explanation of study objectives, procedures, and potential risks. The study protocol was conducted in accordance with the Declaration of Helsinki and approved by the Medical Ethics Committee of Yangpu District Central Hospital (Approval No.: LL-2023-KXJS-004). This trial was prospectively registered at the Chinese Clinical Trial Registry (Registration ID: ChiCTR2200066710) to ensure methodological transparency.

#### 2.1.1 Inclusion criteria

(1) Diagnosis: Defined by the 2020 Chinese Guidelines for Prevention and Treatment of T2DM with≥1 criterion: Classic symptoms (polyuria, polydipsia, weight loss) and random plasma glucose≥11.1 mmol/L; Fasting plasma glucose (FPG) ≥7.0 mmol/L; 2-h postprandial glucose≥11.1 mmol/L during oral glucose tolerance test (OGTT); Glycated hemoglobin (HbA1c) ≥6.5%; (2) Demographics: Age 30–69 years; (3) Glycemic control: FPG ≤ 16.0 mmol/L during preceding 3 months without recurrent hypoglycemia (<2 episodes/month); (4) Sedentary lifestyle: No regular exercise (<2 sessions/week, <30 min/session) in prior 3 months; (5) Disease duration: T2DM duration ≤ 5 years.

#### 2.1.2 Exclusion criteria

(1) Type 1 diabetes, gestational diabetes, or other specific diabetes types; (2) Psychiatric disorders, communication barriers, or motor dysfunction (e.g., hemiplegia, amputation); (3) Acute infections during baseline or intervention periods; (4) Severe diabetic complications (retinopathy, nephropathy, neuropathy, or macrovascular disease).

#### 2.1.3 Sample size estimation

Using G*Power 3.1 with *a priori* analysis for dependent t-tests (two-tailed α=0.05, effect size dz=0.5, power=95%), the minimum required sample size was 54 participants (df=53, noncentrality parameter δ=3.674). Accounting for 20% anticipated attrition, 68 participants were enrolled.

### 2.2 Study design

**Figure 1:**
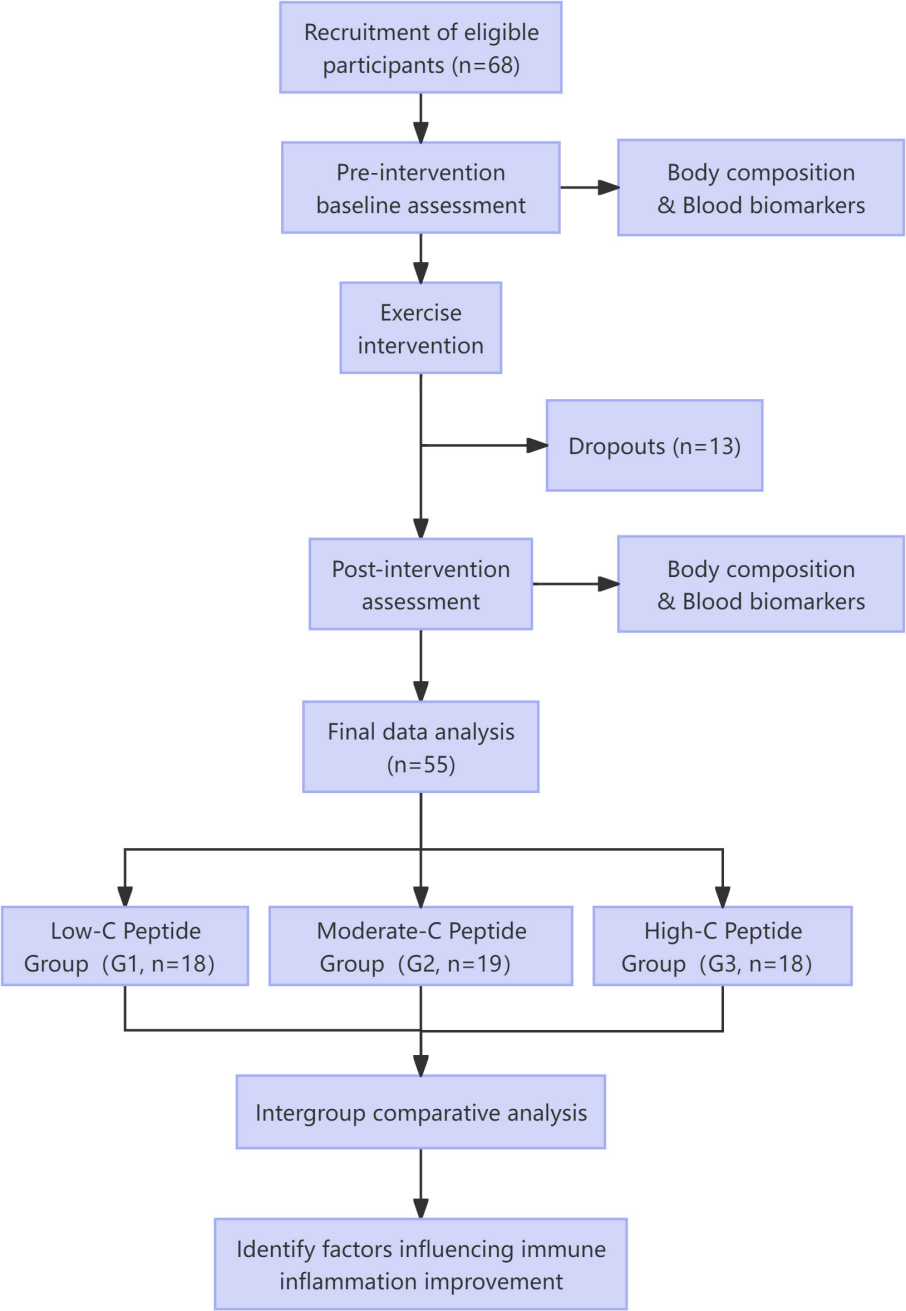
Study flowchart.

This study employed a pre-post controlled design (**Figure 1**). Baseline assessments included demographic data (age, gender), diabetes duration, and medication history. All participants completed a 4-week moderate-intensity combined aerobic and resistance exercise intervention. Venous blood samples were collected pre- and post-intervention for biochemical analyses (FPG, HbA1c, blood lipids, C-peptide, fasting insulin (FINS), neutrophils, lymphocytes, platelets) and calculation of IR indices (HOMA-IR, TyG index) along with β-cell function assessment (HOMA-β). Anthropometric measurements (body weight, BMI, body fat percentage, lean body mass) were concurrently obtained.

### 2.3 Intervention protocol

The exercise intervention was designed as a combined training regimen in strict accordance with the 2022 American College of Sports Medicine (ACSM) guidelines for T2DM management. This integrated program included aerobic, resistance, balance, and flexibility components to elicit comprehensive physiological benefits. Under the supervision of certified exercise rehabilitation specialists, participants underwent a 4-week program consisting of three 60–75-minute sessions per week (schedule: Monday, Wednesday, Friday, 19:00–20:30). The intensity of aerobic exercise was maintained at 50–60% of heart rate reserve, monitored in real-time using Polar H10 chest-strap monitors.

Each session was structured as follows:

1. Warm-up (10 minutes): Dynamic stretching and light aerobic activities to prepare the neuromuscular system.
2. Aerobic Exercise (30 minutes): Treadmill walking or cycling at 50–60% heart rate reserve.
3. Resistance Training (20–25 minutes): Two sets of 10–15 repetitions targeting major muscle groups, using resistance bands and body weight.
4. Balance and Flexibility Cooldown (10 minutes): Static stretching for major muscle groups, combined with balance exercises (e.g., single-leg stands).

Throughout the study, participants maintained their habitual dietary intake. Adherence to the protocol was strictly monitored, and only those who completed at least 80% of the sessions (≥10 out of 12 sessions) were included in the final analysis. Identical assessments were conducted before and after the intervention.

### 2.4 Metabolic parameter calculations

#### 2.4.1 Systemic immune-inflammation index


$$\text{SII}=\frac{\text{Neutrophil}\;\text{count}\;(\times 10^{9}/\text{L})\times \text{Platelet}\;\text{count}\;(\times 10^{9}/\text{L})}{\text{Lymphocyte}\;\text{count}\;(\times 10^{9}/\text{L})}$$

#### 2.4.2 Insulin resistance indices


$$\text{HOMA}-\text{IR}=\frac{\text{Fasting insulin }(\text{μIU}/\text{mL})\times \text{Fasting glucose }(\text{mmol}/\text{L})}{22.5}$$

TyG Index=Ln [Fasting triglycerides (mg/dL)×Fasting glucose (mg/dL)/2]

#### 2.4.3 β-Cell function indices


$$\text{HOMA}-\text{β}=\frac{20\times \text{Fasting insulin }(\text{μIU}/\text{mL})}{\left[\text{Fasting glucose }(\text{mmol}/\text{L})-3.5\right]}$$

### 2.5 Statistical analysis

Data were analyzed using SPSS 27.0 and GraphPad Prism 9.0. Continuous variables underwent normality assessment via Shapiro-Wilk tests, with normally distributed parameters reported as mean ± standard deviation and non-normally distributed variables as median (interquartile range). Within-group pre-post comparisons were performed using paired t-tests for normally distributed data and Wilcoxon signed-rank tests for non-normal distributions. Participants stratified by baseline C-peptide tertiles (Group 1/2/3) were compared using ANCOVA with baseline values as covariates to evaluate intergroup differences in intervention effects, applying Bonferroni correction for multiple comparisons (α = 0.05, two-tailed). Correlation analyses between baseline C-peptide and ΔSII employed Pearson’s or Spearman’s methods based on data distribution. Hierarchical multiple linear regression models were constructed to determine baseline C-peptide’s independent predictive value for ΔSII: Model 1 (unadjusted), Model 2 (adjusted for gender/age), Model 3 (additional adjustment for diabetes duration), and Model 4 (full adjustment including BMI).

## 3 Results

Among the initial cohort of 68 enrolled participants, 10 discontinued the 4-week intervention (<10 attendance sessions) and 3 were excluded due to upper respiratory infections (n=2) or dermatitis (n=1) that could confound hematological measurements. Ultimately, 55 participants completed the intervention with full data collection. Participants were stratified into three groups by baseline fasting C-peptide levels: lowest (Group 1, n=18), intermediate (Group 2, n=19), and highest (Group 3, n=18).

### 3.1 Baseline characteristics

C-peptide levels in Groups 1 and 2 were within normal range (0.8-4.2 ng/mL; Group 1: 1.25 [1.12, 1.34]; Group 2: 2.09 [1.81, 2.83]), while Group 3 levels predominantly exceeded this range (3.92 [3.50, 5.40]). Group 3 demonstrated significantly higher FINS (22.41 [17.32, 32.82]) and HOMA-IR (8.09 [5.15, 10.93]) versus Groups 1-2 (*p*<0.05). The triglyceride-glucose index (TyG) and HOMA-β were also significantly elevated in Group 3 compared to other groups. Additionally, Group 3 exhibited significantly greater body weight, BMI, body fat percentage, lean body mass, and triglycerides (TG) than Group 1 (*p*<0.05). No significant intergroup differences (*p*>0.05) were observed in gender, age, T2D duration, glucose, HbA1c, total cholesterol (TC), high-density lipoprotein cholesterol (HDL-C), low-density lipoprotein cholesterol (LDL-C), or SII, confirming baseline comparability (**Table 1**).

**Table 1:** Baseline characteristics of participants.

Variables	Total	Group 1	Group 2	Group 3	Statistic	*p*
N	55	18	19	18		
male/female (n)	35/20	9/9	14/5	12/6	χ²=2.35	0.309
Age (years)	46.53 ± 11.93	48.33 ± 12.49	49.58 ± 11.85	41.50 ± 10.35	F=2.57	0.087
T2D duration (years)	2.67 ± 1.17	2.56 ± 1.47	2.95 ± 0.81	2.50 ± 1.15	F=0.81	0.451
Weight (kg)	77.39 ± 16.19	66.94 ± 13.65^a^	79.22 ± 14.47^b^	85.91 ± 15.10^b^	F=8.02	<.001
BMI	27.50 ± 4.91	24.55 ± 5.10^a^	28.04 ± 4.43^ab^	29.89 ± 3.79^b^	F=6.64	0.003
BFP (%)	29.99 ± 7.76	26.77 ± 8.28^a^	30.10 ± 7.19^ab^	33.10 ± 6.82^b^	F=3.25	0.047
LBM (kg)	50.72 ± 10.77	45.35 ± 7.19^a^	51.59 ± 10.57^ab^	55.17 ± 12.10^b^	F=4.31	0.019
HbA1c (%)	6.90 (6.25, 7.50)	6.65 (6.12, 7.38)	7.00 (6.45, 7.45)	7.10 (6.50, 7.88)	χ²=1.76	0.415
FPG (mmol/L)	6.52 (5.63, 8.04)	6.42 (5.84, 7.10)	6.45 (5.44, 8.21)	6.84 (6.13, 8.78)	χ²=0.70	0.703
C Peptide (ng/mL)	2.09 (1.38, 3.44)	1.25 (1.12, 1.34)^a^	2.09 (1.81, 2.83)^a^	3.92 (3.50, 5.40)^b^	χ²=48.01	<.001
FINS (μIU/ml)	9.56 (5.17, 16.91)	5.00 (3.97, 6.05)^a^	8.97 (6.38, 11.46)^a^	22.41 (17.32, 32.82)^b^	χ²=31.77	<.001
HOMA-IR	2.51 (1.45, 5.48)	1.45 (1.14, 1.95)^a^	2.51 (1.60, 3.36)^a^	8.09 (5.15, 10.93)^b^	χ²=29.23	<.001
HOMA-β	57.47 (31.78, 125.82)	32.70 (20.52, 43.70) ^a^	45.27 (31.78, 104.40) ^a^	128.99 (74.84, 192.36) ^b^	χ²=22.93	<.001
TyG Index	9.05 ± 0.58	8.80 ± 0.46 ^a^	8.93 ± 0.44 ^a^	9.43 ± 0.65 ^b^	F=7.24	0.002
TC (mmol/L)	5.26 ± 1.19	5.19 ± 1.25	5.26 ± 1.20	5.32 ± 1.19	F=0.05	0.947
TG (mmol/L)	1.62 (1.10, 1.99)	1.15 (0.92, 1.62)^a^	1.52 (1.11, 1.78)^a^	2.01 (1.66, 2.79)^b^	χ²=14.38	<.001
HDL-C (mmol/L)	1.33 ± 0.32	1.39 ± 0.29	1.41 ± 0.38	1.19 ± 0.22	F=2.82	0.069
LDL-C (mmol/L)	3.29 ± 0.79	3.15 ± 0.93	3.14 ± 0.71	3.59 ± 0.66	F=1.94	0.154
NC (10^9/L)	3.86 ± 1.07	3.96 ± 1.29	4.07 ± 0.97	3.54 ± 0.88	F=1.30	0.282
PC (10^9/L)	251.71 ± 74.23	263.33 ± 51.13	234.11 ± 101.20	258.67 ± 59.18	F=0.83	0.442
LC (10^9/L)	2.13 (1.79, 2.44)	1.96 (1.68, 2.38)	2.01 (1.79, 2.41)	2.29 (2.09, 2.50)	χ²=2.34	0.310
SII Index	440.56 ± 177.44	495.20 ± 167.70	414.84 ± 145.74	413.08 ± 211.42	F=1.28	0.286

Data are presented as mean ± standard deviation (SD) for normally distributed continuous variables, and as median (interquartile range) for non-normally distributed continuous variables. Categorical variables are presented as counts.

Statistical tests for group comparisons: One-Way ANOVA (reporting F value) was used for normally distributed continuous variables; the Kruskal-Wallis H test (reporting χ² value) was used for non-normally distributed continuous variables; the chi-squared test (reporting χ² value) was used for categorical variables.

The lowercase letters (e.g., “a”, “b”, “ab”) in the table indicate the results of pairwise comparisons between groups. Groups sharing at least one identical letter are not statistically different (e.g., Group 1 (“a”) and Group 2 (“ab”) share the letter “a”, indicating no significant difference). Conversely, groups without any shared letters (e.g., Group 1 (“a”) and Group 3 (“b”) are significantly different.

BMI, Body Mass Index; BFP, Body Fat Percentage; LBM, Lean Body Mass; HbA1c, Glycated Hemoglobin; FPG, Fasting Plasma Glucose; FINS, Fasting Insulin; HOMA-IR, Homeostatic Model Assessment of Insulin Resistance; TC, Total Cholesterol; TG, Triglycerides; HDL-C, High-Density Lipoprotein Cholesterol; LDL-C, Low-Density Lipoprotein Cholesterol; NC, Neutrophil Count; PC, Platelet Count; LC, Lymphocyte Count; SII, Systemic Immune-Inflammation Index.

### 3.2 Pre-post intervention outcomes

Following a four-week exercise intervention, the baseline measures were reassessed. Comparisons between pre- and post-intervention are detailed in **Table 2**.

**Table 2:** Changes in outcome measures before and after exercise intervention by group.

Variables	Assessment	Group1(n=18)	*p*	Group2(n=19)	*p*	Group3(n=18)	*p*
Weight (kg)	pre	66.94 ± 13.65	< 0.01**	79.22 ± 14.47	< 0.01**	85.91 ± 15.10	ns
	post	65.18 ± 12.33		77.55 ± 13.83		85.14 ± 15.66	
BMI	pre	24.55 ± 5.10	< 0.05*	28.04 ± 4.43	< 0.01**	29.89 ± 3.79	ns
	post	23.91 ± 4.57		27.43 ± 4.20		29.61 ± 3.70	
BFP (%)	pre	26.77 ± 8.28	< 0.01**	30.10 ± 7.19	< 0.01**	33.10 ± 6.82	ns
	post	25.43 ± 8.28		28.84 ± 7.30		32.23 ± 6.53	
LBM (kg)	pre	45.35 ± 7.19	ns	51.59 ± 10.57	ns	55.17 ± 12.10	ns
	post	45.27 ± 7.85		51.65 ± 10.52		55.07 ± 12.76	
HbA1c (%)	pre	6.65(6.10, 7.40)	< 0.05*	7.00(6.40, 7.50)	< 0.01**	7.10(6.45, 8.08)	< 0.001***
	post	6.25(5.90, 6.78)		6.40(5.90, 6.70)		5.85(5.40, 6.78)	
FPG (mmol/L)	pre	6.42(5.76, 7.29)	< 0.05*	6.45(5.39, 8.33)	ns	6.85(6.11, 9.16)	= 0.01*
	post	6.01(5.28, 7.00)		6.25(5.65, 6.68)		5.96(5.55, 6.88)	
C peptide (ng/mL)	pre	1.25(1.08, 1.36)	ns	2.09(1.76, 2.94)	< 0.01**	3.92(3.47, 5.61)	< 0.01**
	post	1.50(1.08, 2.00)		1.88(1.58, 2.21)		3.59(2.58, 4.61)	
FINS (μIU/ml)	pre	5.00(3.65, 6.10)	ns	8.97(5.71, 11.56)	< 0.05*	22.42(16.23, 34.88)	< 0.05*
	post	5.56(3.52, 7.79)		7.73(5.12, 9.41)		17.37(12.71, 24.16)	
HOMA-IR	pre	1.45(1.08, 2.02)	ns	2.51(1.40, 3.61)	< 0.05*	8.09(4.91, 12.15)	< 0.01**
	post	1.66(0.87, 2.27)		1.98(1.42, 3.04)		5.30(3.49, 7.60)	
HOMA-β	pre	32.70(19.29, 47.84)	ns	45.27(31.55, 122.96)	ns	128.99(71.84, 209.42)	ns
	post	40.78(29.91, 51.44)		48.34(36.01,68.94)		146.08(85.70, 191.63)	
TyG Index	pre	8.80 ± 0.46	ns	8.93 ± 0.44	< 0.05*	9.43 ± 0.65	< 0.01**
	post	8.72 ± 0.45		8.72 ± 0.50		9.17 ± 0.62	
TC (mmol/L)	pre	5.19 ± 1.25	ns	5.26 ± 1.20	ns	5.32 ± 1.19	ns
	post	4.95 ± 1.33		5.06 ± 1.23		5.13 ± 0.57	
TG (mmol/L)	pre	1.15(0.87, 1.69)	ns	1.52(1.07, 1.80)	< 0.05*	2.01(1.64, 2.98)	ns
	post	1.34(1.07, 1.59)		1.17(0.92, 1.82)		1.62(1.42, 2.90)	
HDL-C (mmol/L)	pre	1.39 ± 0.29	ns	1.41 ± 0.38	< 0.01**	1.19 ± 0.22	ns
	post	1.41 ± 0.35		1.51 ± 0.35		1.19 ± 0.21	
LDL-C (mmol/L)	pre	3.15 ± 0.93	ns	3.15 ± 0.71	ns	3.59 ± 0.66	< 0.01**
	post	3.04 ± 1.01		3.10 ± 0.77		3.14 ± 0.52	
NC (10^9/L)	pre	3.96 ± 1.29	< 0.05*	4.07 ± 0.97	ns	3.54 ± 0.88	< 0.05*
	post	3.42 ± 0.97		3.76 ± 0.79		3.84 ± 0.91	
PC (10^9/L)	pre	263.33 ± 51.13	ns	234.11 ± 101.20	ns	258.67 ± 59.18	ns
	post	256.72 ± 56.93		236.60 ± 87.72		266.00 ± 57.11	
LC (10^9/L)	pre	1.97(1.66, 2.41)	ns	2.01(1.79, 2.45)	ns	2.29(2.06, 2.62)	ns
	post	1.96(1.64, 2.39)		2.11(1.75, 2.86)		2.14(1.86, 2.81)	
SII Index	pre	495.20 ± 167.70	< 0.05*	414.84 ± 145.74	ns	413.08 ± 211.42	ns
	post	429.63 ± 129.34		390.25 ± 124.53		464.13 ± 182.19	

Data are presented as mean ± standard deviation (SD) for normally distributed variables, and as median (interquartile range) for non-normally distributed variables.

Within-group comparisons (pre vs. post) were performed using the Paired t-test for normally distributed data and the Wilcoxon signed-rank test for non-normally distributed data.

Significance markers: *p < 0.05, **p < 0.01, ***p < 0.001; “ns” denotes non-significance (*p* > 0.05).

#### 3.2.1 Anthropometric measures

Significant improvements in body weight, BMI, and body fat percentage were observed in both Group 1 and Group 2 compared to baseline (*p* < 0.05). However, Group 3 showed no significant changes in these parameters (*p* > 0.05). Notably, no significant improvements in lean body mass were observed in any group (*p* > 0.05).

#### 3.2.2 Glycemic control measures

HbA1c showed significant improvement from baseline in all three groups (*p* < 0.05). FPG significantly improved only in Group 1 and Group 3 (*p* < 0.05), with no significant change observed in Group 2 (*p* > 0.05).

#### 3.2.3 Insulin resistance indices

Fasting C-peptide demonstrated a non-significant tendency toward increase in Group 1 (*p* > 0.05), remaining within normal range. Conversely, significant decreases were observed in Group 2 and Group 3 (*p* < 0.05). FINS and HOMA-IR paralleled this pattern of change. No significant changes in HOMA-β were observed in any group.

#### 3.2.4 Lipid profile measures

TC showed a non-significant decreasing tendency across all groups (*p* > 0.05). TG decreased significantly only in Group 2 (*p* < 0.05), with no significant changes in Group 1 or Group 3. HDL-C levels significantly improved in Group 2 (*p* < 0.05) but remained unchanged in Group 1 and Group 3 (*p* > 0.05). LDL-C levels decreased significantly only in Group 3 (*p* < 0.05), while Groups 1 and 2 showed non-significant reducing trends (*p* > 0.05).

#### 3.2.5 Inflammatory markers

Changes in SII and its components (neutrophils, platelets, lymphocytes) were analyzed post-intervention. SII decreased significantly only in Group 1 (*p* < 0.05), with no significant improvements in Group 2 or Group 3 (*p* > 0.05). Neutrophil count significantly decreased in Group 1 (*p* < 0.05), showed no change in Group 2 (*p* > 0.05), but significantly increased in Group 3 (*p* < 0.05). Neither platelet count nor lymphocyte count showed significant improvements in any group (*p* > 0.05).

### 3.3 Differential changes across groups

**Figure 2:**
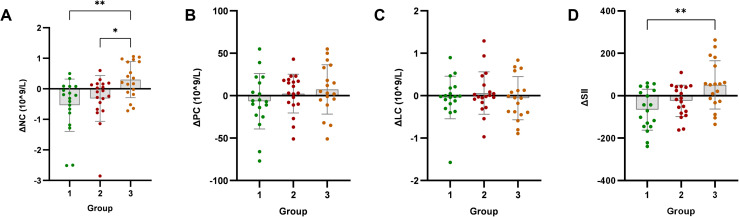
Analysis of immune-inflammatory indicators after exercise intervention. Bar plots with overlaid individual data points display the mean change from baseline (Δ, calculated as post-intervention value minus baseline value) for **(A)** neutrophil count (NC), **(B)** platelet count (PC), **(C)** lymphocyte count (LC), and **(D)** systemic immune-inflammation index (SII) across the three groups stratified by baseline C-peptide: Group 1 (n=18), Group 2 (n=19), and Group 3 (n=18). Individual dots represent each participant’s response. Asterisks denote significant within-group changes (paired t-test or Wilcoxon test): *p < 0.05, **p < 0.01, ***p < 0.001; the absence of asterisks indicates non-significance.

**Figure 3:**
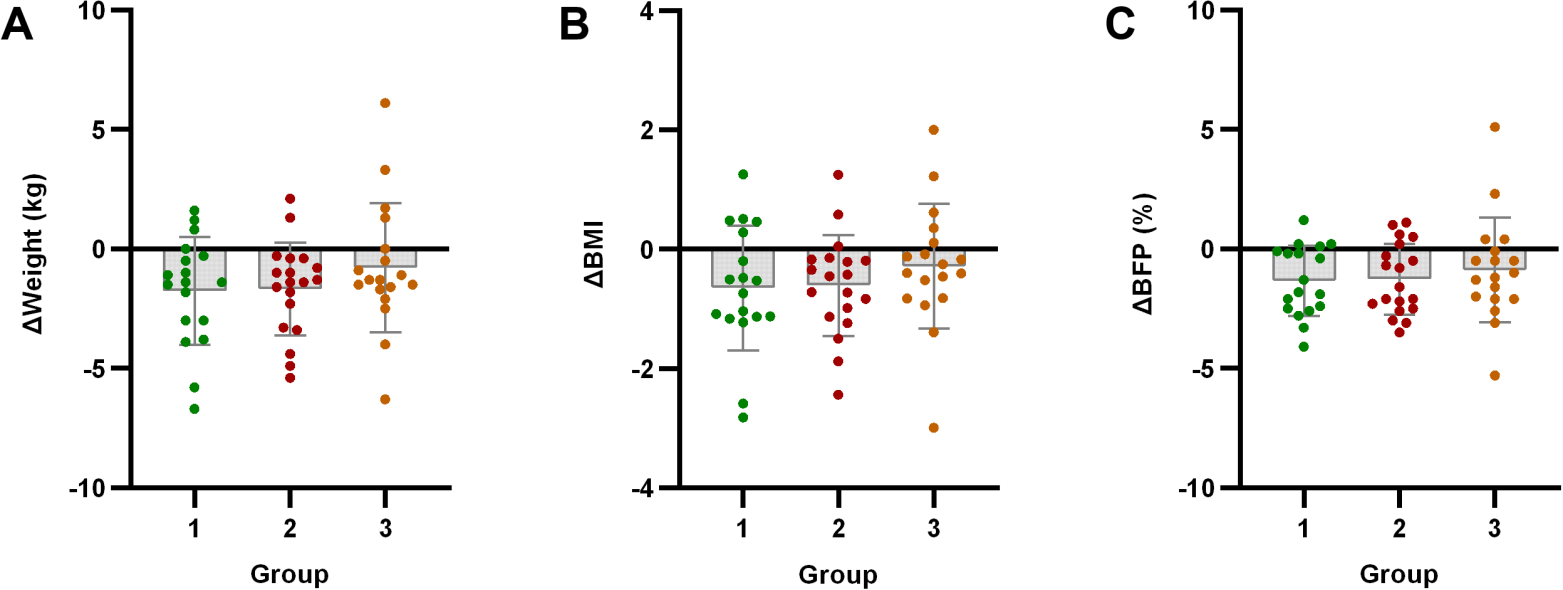
Analysis of body composition indicators after exercise intervention. Bar plots with overlaid individual data points display the mean change from baseline (Δ, calculated as post-intervention value minus baseline value) for **(A)** Weight, **(B)** body mass index (BMI), and **(C)** body fat percentage (BFP) across the three groups stratified by baseline C-peptide: Group 1 (n=18), Group 2 (n=19), and Group 3 (n=18). Individual dots represent each participant’s response. Asterisks denote significant within-group changes (paired t-test or Wilcoxon test): *p < 0.05, **p < 0.01, ***p < 0.001; the absence of asterisks indicates non-significance.

**Figure 4:**
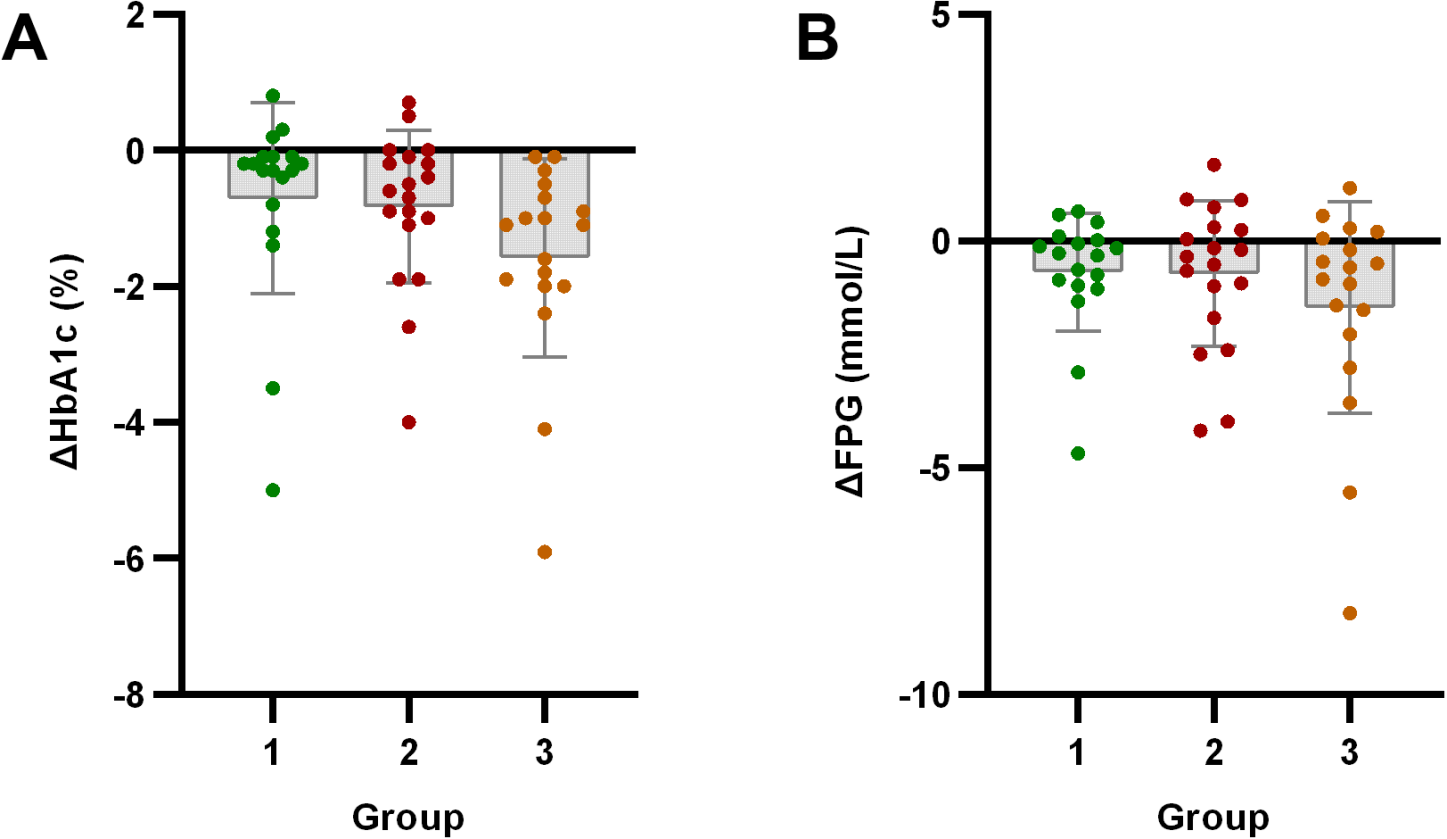
Analysis of glucose metabolism indicators after exercise intervention. Bar plots with overlaid individual data points display the mean change from baseline (Δ, calculated as post-intervention value minus baseline value) for **(A)** HbA1c and **(B)** Fasting Plasma Glucose (FPG) across the three groups stratified by baseline C-peptide: Group 1 (n=18), Group 2 (n=19), and Group 3 (n=18). Individual dots represent each participant’s response. Asterisks denote significant within-group changes (paired t-test or Wilcoxon test): *p < 0.05, **p < 0.01, ***p < 0.001; the absence of asterisks indicates non-significance.

**Figure 5:**
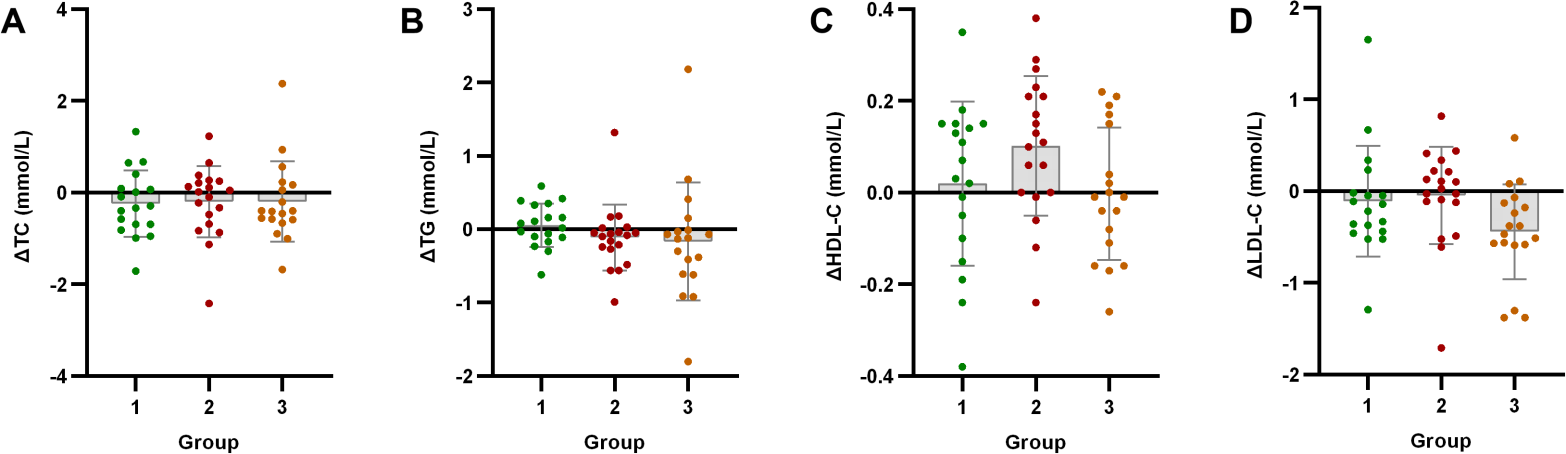
Analysis of lipid metabolism indicators after exercise intervention. Bar plots with overlaid individual data points display the mean change from baseline (Δ, calculated as post-intervention value minus baseline value) for **(A)** total cholesterol (TC), **(B)** triglycerides (TG), **(C)** high-density lipoprotein cholesterol (HDL-C), and **(D)** low-density lipoprotein cholesterol (LDL-C) across the three groups stratified by baseline C-peptide: Group 1 (n=18), Group 2 (n=19), and Group 3 (n=18). Individual dots represent each participant’s response. Asterisks denote significant within-group changes (paired t-test or Wilcoxon test): *p < 0.05, **p < 0.01, ***p < 0.001; the absence of asterisks indicates non-significance.

Intergroup analysis revealed significant differential changes in neutrophil count and SII following the 4-week intervention (*p* < 0.05). Groups with milder baseline IR (Groups 1 and 2) demonstrated improvements in immune-inflammatory markers. Conversely, Group 3 - characterized by significant baseline IR - showed no improvement in SII, moreover exhibiting deterioration through significantly elevated neutrophil counts (*p* < 0.05) (**Figure 2**). No statistically significant intergroup differences were observed in anthropometric parameters or glucolipid metabolic measures (*p* > 0.05) (**Figures 3**–**5**).

### 3.4 Association between baseline C-peptide and SII improvement

**Figure 6:**
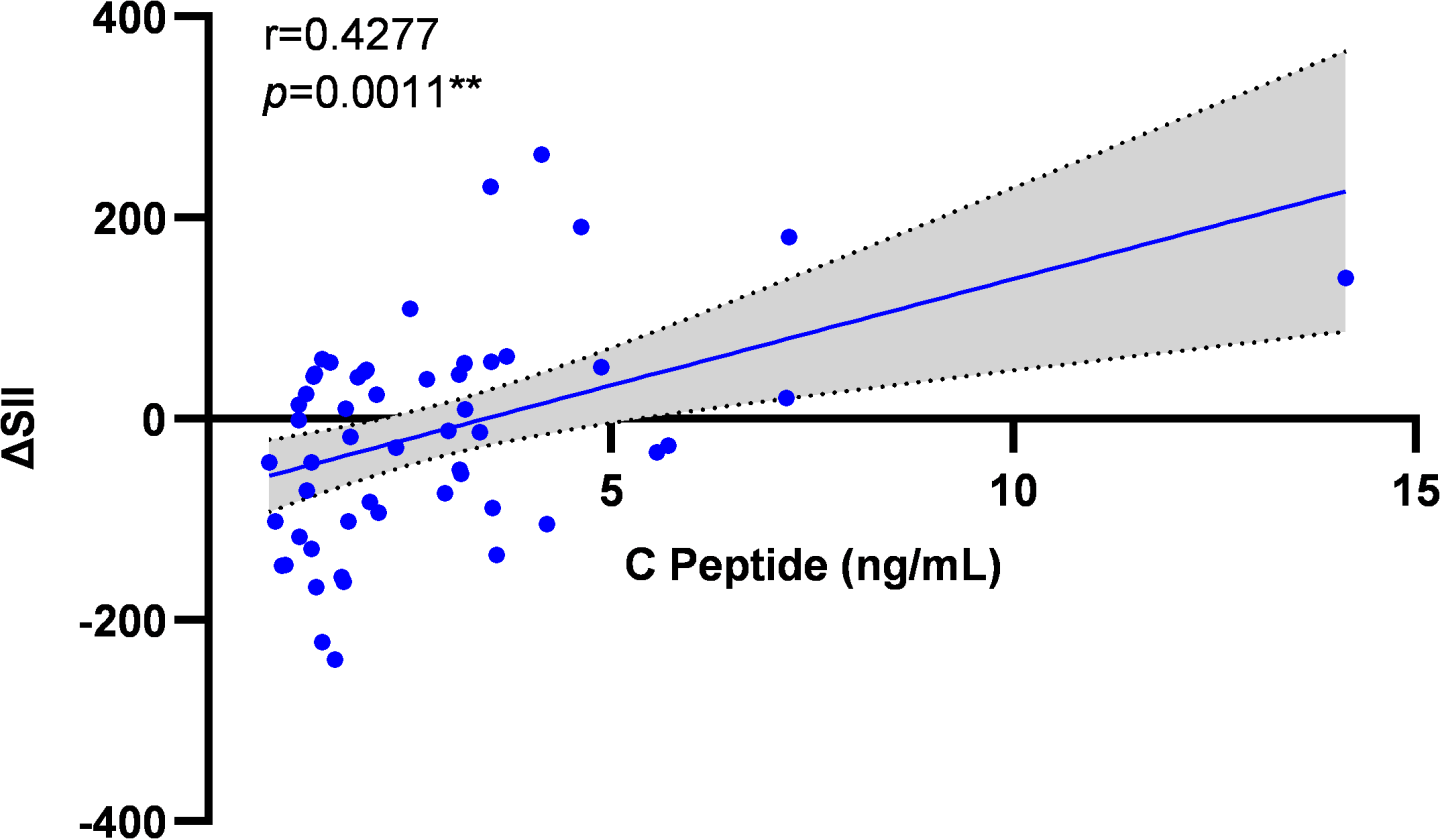
Correlation analysis between baseline C-peptide and ΔSII improvement. Scatter plot illustrating the relationship between baseline C-peptide levels and the change in SII (ΔSII, calculated as post-intervention value minus baseline value) following the exercise intervention across all participants (N = 55). The solid line represents the line of best fit from the Spearman correlation analysis. Correlation coefficient (r = 0.428) and significance (*p* = 0.001) are indicated.

Baseline C-peptide levels showed significant positive correlation with post-intervention systemic immune-inflammation index improvement (ΔSII) (r = 0.428, *p* = 0.001) (**Figure 6**).

### 3.5 Baseline C-peptide as independent predictor of ΔSII

Multiple linear regression confirmed baseline C-peptide as an independent predictor of ΔSII across sequential adjustment models: unadjusted (B = 21.14, SE = 6.02, *p* < 0.001), gender/age-adjusted (B = 21.94, SE = 6.42, *p* < 0.01), with additional diabetes duration adjustment (B = 21.17, SE = 6.68, *p* < 0.01), and full adjustment including BMI (B = 19.85, SE = 6.92, *p* < 0.01). All covariates demonstrated nonsignificant contributions (gender: *p* > 0.05; age: *p* > 0.05; T2D duration: *p* > 0.05; BMI: *p* > 0.05) (**Table 3**).

**Table 3:** Multivariable linear regression analysis of baseline C-peptide and ΔSII improvement.

Variable	Model 1	Model 2	Model 3	Model 4
C-peptide	21.14***	21.94**	21.17**	19.85**
	(6.02)	(6.42)	(6.68)	(6.92)
Gender	—	8.43	9.59	12.47
		(27.91)	(28.23)	(28.59)
Age	—	0.36	0.16	0.57
		(1.16)	(1.24)	(1.35)
T2D duration	—	—	5.87	5.17
			(12.49)	(12.58)
BMI	—	—	—	2.50
				(3.23)
Constant	-72.20**	-102.61	-108.50	-194.89
	(21.22)	(76.26)	(77.87)	(136.28)

The dependent variable in all models is ΔSII (calculated as post-intervention SII minus baseline SII).

Unstandardized regression coefficients (B) are reported with their standard errors (SE) in parentheses.

The analysis included all participants who completed the study (N = 55).

Model 1: Unadjusted; Model 2: Adjusted for gender, age; Model 3: Adjusted for gender, age, T2D duration; Model 4: Adjusted for gender, age, T2D duration, BMI

Significance levels: *p < 0.05, **p < 0.01, ***p < 0.001.

## 4 Discussion

This study stratified early-stage T2DM patients (disease duration ≤5 years, fasting C-peptide >0.8 ng/mL) by baseline C-peptide levels, justified by its correlation with IR and unique advantages: 1) Resilience to β-cell functional heterogeneity; 2) Superior stability over HOMA-IR assays; 3) Methodological extensibility to insulin-treated populations. Baseline validation confirmed group efficacy—the high C-peptide cohort (Group 3) concurrently exhibited peak HOMA-IR, TyG index, and FINS. Baseline characteristics showed that Group 3 patients’ severe IR was associated with worse metabolic dysregulation. This group not only had significantly higher IR markers (FINS, HOMA-IR, TyG index) and β-cell function marker HOMA-β than low/moderate groups (Group 1/Group 2), but also exhibited greater anthropometric measures (weight, BMI, body fat percentage, lean body mass). Notably, while no significant differences existed in baseline fasting glucose, HbA1c, TC, HDL-C, LDL-C, or SII among groups, Group 3 had significantly elevated baseline TG. This aligns with expectations since worsened IR and obesity often accompany hypertriglyceridemia (35).

This study implemented a 4-week combined aerobic-resistance training intervention. The selection of this intervention duration was based on a comprehensive consideration of prior evidence and study feasibility. Existing literature indicates that a structured 4-week exercise program is sufficient to induce significant early improvements in insulin sensitivity and inflammatory markers in individuals with T2DM (36–38). Furthermore, preclinical studies have confirmed that a 4-week training period can induce significant metabolic adaptations, including improved glucose tolerance, in relevant animal models (39). From a practical perspective, and as an intensively supervised pilot study, this shorter duration was crucial for ensuring high participant adherence, thereby controlling dropout rates and generating high-quality evidence for the early effects of exercise intervention. Current evidence supports that combined aerobic-resistance exercise synergistically optimizes metabolic benefits in T2DM (40), further justifying our protocol design. Post-intervention, we excluded patients developing upper respiratory infections or dermatitis during the study to ensure SII changes primarily reflected exercise effects. Key findings emerged from group analyses:

Anthropometric improvements correlated with baseline IR severity: Despite Group 3 (high-IR) having the highest baseline weight, BMI, and body fat%, its post-intervention improvements were significantly lower than Group 1/Group 2 (low/moderate-IR). Group 1/Group 2 showed significant anthropometric improvements, while Group 3 had no statistically significant changes. This suggests severe pre-intervention IR may hinder exercise’s positive effects on obesity-related body composition. Importantly, exercise preserved lean body mass (Δlean mass: *p*>0.05 in all groups), contrasting sharply with lean mass loss from pharmacological/dietary interventions, which holds clinical significance for delaying sarcopenia in T2DM.

Limited inflammatory improvement in high baseline-IR group: Consistent with attenuated anthropometric improvements, Group 3’s SII showed no significant improvement post-intervention, instead trending upward. This indicates baseline high-IR may compromise exercise-induced systemic anti-inflammatory effects. These results suggest that T2DM patients with severe IR experience a hyperinflammatory phase during early exercise intervention, during which anthropometric improvements are also limited. Further analysis revealed SII reduction primarily stemmed from decreased neutrophil counts (*p*<0.05), not platelet reduction or lymphocyte increase, confirming exercise improves immune-inflammation mainly through neutrophil modulation. Several studies report neutrophils’ predictive value for T2DM remission. For instance, Bonaventura et al. (2019) found NLR ≤1.97 predicted 5-year T2DM remission post-bariatric surgery (41). Zubiaga et al. (2020) showed preoperative NLR correlated with 5-year postoperative anthropometric/glycemic outcomes and complete T2DM remission (42). Lower baseline neutrophils predicted higher T2DM remission likelihood after Mediterranean diet interventions (43).

The observed improvement in SII, particularly the reduction in neutrophil count, can be interpreted within the established biological framework of exercise-induced anti-inflammatory effects. The SII reflects a balance between pro-inflammatory (neutrophils, platelets) and immuno-regulatory (lymphocytes) forces. Its decrease signifies a systemic shift toward a less inflammatory state. This shift is likely mediated through multiple mechanisms: 1) Exercise reduces visceral adipose tissue mass, a primary site of pro-inflammatory cytokine production (e.g., TNF-α, IL-1β) due to infiltrated M1 macrophage (44). 2) Contracting skeletal muscle acts as an endocrine organ, releasing myokines such as IL-6, which in the context of exercise stimulates the production of anti-inflammatory cytokines like IL-10 and inhibits TNF-α release (45, 46). 3) Regular exercise can dampen the activation of innate immune signaling pathways, including Toll-like receptors (TLRs), thereby reducing downstream NF-κB-driven inflammation (47, 48). It is important to note that while our study demonstrates modulation of systemic cellular inflammation via SII, this composite marker does not resolve specific downstream pathways. Future research should directly measure the cytokine milieu (e.g., IL-1β, IL-6, TNF-α, IL-10) and signaling molecules to fully elucidate the precise mechanisms at play.

Glycemic improvements independent of weight loss/anti-inflammatory effects: Strikingly, despite limited anthropometric and inflammatory improvements, Group 3 showed significant glycemic control improvements (fasting glucose, HbA1c). This clearly demonstrates exercise’s glycemic benefits can occur independently of significant weight/fat loss or systemic inflammation improvement. This finding is clinically crucial, as it underscores that patients with severe IR can achieve meaningful glycemic control through exercise, even before substantial weight loss or systemic anti-inflammatory effects are evident.

We propose that the mechanism by which high IR hinders weight loss relates to inflammation: Baseline high-IR patients showed insignificant weight/fat reduction and elevated SII (not improvement) during early exercise (4 weeks), while IR itself improved significantly. This suggests high-IR status or its metabolic environment (e.g., adipose tissue dysfunction, signaling abnormalities) may inhibit exercise-induced fat loss. A prediabetes study found only 31% of high-risk phenotype patients (with severe IR and NAFLD) achieved normal glucose after lifestyle intervention, versus 67% in low-risk group (49). This indicates severe-IR patients require longer interventions for equivalent efficacy—a potential explanation for differential exercise-induced fat loss in patients with varying IR severity. Although exercise improved insulin sensitivity in these patients, it might be insufficient to overcome short-term fat loss barriers, possibly linked to transiently elevated inflammation and delayed fat mobilization. However, a Chilean study reported no difference in non-response rates for HOMA-IR improvements after 10-week HIIT across IR strata (50), potentially due to HIIT’s unique anaerobic effects.

We further observed independence of glycemic improvements: Group 3’s significant glycemic improvements occurred despite limited anthropometric/inflammatory changes, confirming exercise’s glycemic benefits can manifest independently. Supporting evidence comes from other IR populations: Obese children showed improved insulin sensitivity after 8-week exercise without significant lean mass/abdominal fat changes via DEXA. Similarly, 1-week intense aerobic exercise improved glycemic parameters without weight change by enhancing peripheral glucose uptake and suppressing hepatic glucose production (51).

This study also reveals complex inflammation-hyperglycemia interactions: Chronic inflammation critically drives T2DM (52, 53), while hyperglycemia exacerbates inflammation via “glucotoxicity” (54). Although prior studies reported concurrent improvements in glucose (FINS, FBG) and inflammation (TNF-α, IL-6, hs-CRP) after 4-week aerobic exercise (55), we observed “glycemic improvement without synchronized inflammation improvement” in Group 3. This suggests exercise may prioritize glucoregulatory pathways over anti-inflammatory modulation in high-IR patients, requiring longer duration or higher intensity for anti-inflammatory effects. A plausible hypothesis: During early intervention (≤4 weeks), exercise activates immune pathways (e.g., acute-phase response) in high-IR individuals, partially counteracting anti-inflammatory effects. Heterogeneous findings across exercise modalities (aerobic (33, 56, 57)/resistance (32, 33, 58–61)/combined (32, 33, 61) may stem from differences in baseline IR severity, intervention parameters (duration/frequency/intensity), assessment timing, and inflammatory biomarkers.

In summary, baseline IR severity critically modulates exercise-induced metabolic and anti-inflammatory responses in T2DM. High-IR significantly attenuates body composition optimization and systemic inflammation mitigation, necessitating IR-stratified exercise prescriptions. Limitations of this study should be acknowledged. First, although key parameters of exercise performance, such as aerobic capacity (e.g., VO_2_max) and muscular strength, were monitored throughout the intervention, they were not included in the present analysis, as the primary focus of this manuscript was to elucidate the direct relationships between the exercise intervention, changes in body composition, and systemic inflammatory/metabolic biomarkers. We believe that exploring the mediating role of fitness gains constitutes a substantial research question that warrants a separate, dedicated investigation. Nevertheless, the absence of this correlation analysis limits our ability to fully delineate whether the observed benefits were directly mediated through improvements in fitness. Additionally, limitations include: Limited sample size potentially affecting subgroup power; 4-week duration possibly insufficient to observe long-term inflammatory trajectories; SII as a composite marker without downstream pathway resolution. Future studies should: Enlarge high-IR subgroups; Extend interventions to 12–24 weeks; Investigate mechanisms (adipose tissue inflammation, immune cell subsets, signaling pathways); Develop personalized high-intensity/long-duration/nutrition-combined strategies to concurrently achieve glycemic control, weight/fat reduction, and anti-inflammatory goals.

## Data Availability

The original contributions presented in the study are included in the article/supplementary material. Further inquiries can be directed to the corresponding authors.
